# Determination of biomarker candidates for the placenta accreta spectrum by plasma proteomic analysis

**DOI:** 10.1038/s41598-024-53324-5

**Published:** 2024-02-02

**Authors:** Rauf Melekoglu, Seyma Yasar, Cemil Colak, Murat Kasap, Umran Karabulut Dogan, Saim Yologlu, Ercan Yilmaz, Sherif Shazly

**Affiliations:** 1 Department of Obstetrics and Gynecology, Faculty of Medicine, Inonu University, 44280 Malatya, Turkey; 2 Department of Biostatistics and Medical Informatics, Faculty of Medicine, Inonu University, 44280 Malatya, Turkey; 3 Department of Medical Biology, Faculty of Medicine, Kocaeli University, Kocaeli, Turkey; 4 Clinic of Obstetrics and Gynecology, Malatya Education and Research Hospital, Malatya, Turkey; 5 Department of Obstetrics and Gynecology, Leeds Teaching Hospitals NHS Trust, Leeds, UK

**Keywords:** Biomarkers, Medical research

## Abstract

Placenta accreta spectrum (PAS) presents a significant obstetric challenge, associated with considerable maternal and fetal-neonatal morbidity and mortality. Nevertheless, it is imperative to acknowledge that a noteworthy subset of PAS cases remains undetected until the time of delivery, thereby contributing to an augmented incidence of morbidity among the affected individuals. The delayed identification of PAS not only hinders timely intervention but also exacerbates the associated health risks for both the maternal and fetal outcomes. This underscores the urgency to innovate strategies for early PAS diagnosis. In this study, we aimed to explore plasma proteins as potential diagnostic biomarkers for PAS. Integrated transcriptome and proteomic analyses were conducted to establish a novel diagnostic approach. A cohort of 15 pregnant women diagnosed with PAS and delivering at Inonu University Faculty of Medicine between 01/04/2021 and 01/01/2023, along with a matched control group of 15 pregnant women without PAS complications, were enrolled. Plasma protein identification utilized enzymatic digestion and liquid chromatography-tandem mass spectrometry techniques. Proteomic analysis identified 228 plasma proteins, of which 85 showed significant differences (*P* < 0.001) between PAS and control cases. We refined this to a set of 20 proteins for model construction, resulting in a highly accurate classification model (96.9% accuracy). Notable associations were observed for proteins encoded by P01859 (Immunoglobulin heavy constant gamma 2), P02538 (Keratin type II cytoskeletal 6A), P29622 [Kallistatin (also known as Serpin A4)], P17900 (Ganglioside GM2 activator Calmodulin-like protein 5), and P01619 (Immunoglobulin kappa variable 3–20), with fold changes indicating their relevance in distinguishing PAS from control groups. In conclusion, our study has identified novel plasma proteins that could serve as potential biomarkers for early diagnosis of PAS in pregnant women. Further research and validation in larger PAS cohorts are necessary to determine the clinical utility and reliability of these proteomic biomarkers for diagnosing PAS.

## Introduction

Placenta accreta spectrum (PAS) represents a significant obstetric complication associated with substantial maternal and fetal-neonatal morbidity and mortality^1^. Early diagnosis of PAS is crucial to achieve favorable obstetric and perinatal outcomes. However, precise prenatal diagnosis of PAS is challenging, and current imaging techniques may not always provide definitive conclusions^2^. Consequently, a considerable proportion of PAS cases remain undiagnosed until delivery, leading to increased morbidity among affected individuals. Therefore, there is urgent need to establish new contemporary paradigms for early and accurate diagnosis of women with suspected PAS.

Previous research has investigated potential biomarkers for PAS, including angiogenic markers, aneuploidy serum analysis, and fetal fraction obtained from noninvasive prenatal screening^3^. These tests have been proposed based on our current understanding of the pathogenesis of PAS, which may involve factors such as the absence of the decidual or basal layer, loss of the normal subdecidual myometrium layers, abnormal maternal vascularization, and excessive invasion of extravillous trophoblasts^4,5^. Nevertheless, there is currently no clinically reliable blood or urine biomarker for PAS, possibly since the precise underlying mechanisms of PAS remain incompletely understood.

Advancements in next-generation sequencing technology have enabled comprehensive bioinformatic analyses, offering a multi-omics perspective to understand disease-associated biological samples. While transcriptomic analysis provides insights into gene expression, it does not fully capture the complex post-translational control mechanisms that govern cellular function. Therefore, integrated transcriptome and proteome analysis has emerged as a powerful approach to investigate gene expression regulation for advancing our understanding of complex diseases, and it holds particular significance in the context of PAS^6^. Proteomics, the large-scale study of proteins expressed by a cell, tissue, or organism, offers a unique opportunity to elucidate the intricate molecular landscape associated with PAS. By systematically analyzing the entire complement of proteins present in biological samples, proteomic approaches can provide valuable insights into the specific protein signatures associated with PAS. These protein signatures, reflecting alterations in expression levels, post-translational modifications, and interactions, have the potential to serve as distinctive biomarkers for early and accurate diagnosis.

While bioinformatic studies have the potential to shed light on the pathophysiological mechanisms of abnormally invasive placenta and identify protein biomarkers for diagnosis, there is currently a scarcity of research in this crucial area. The primary objective of this study is to address this gap by identifying potential protein biomarkers for PAS diagnosis through comprehensive proteomic analysis.

## Results

**Figure 1:**
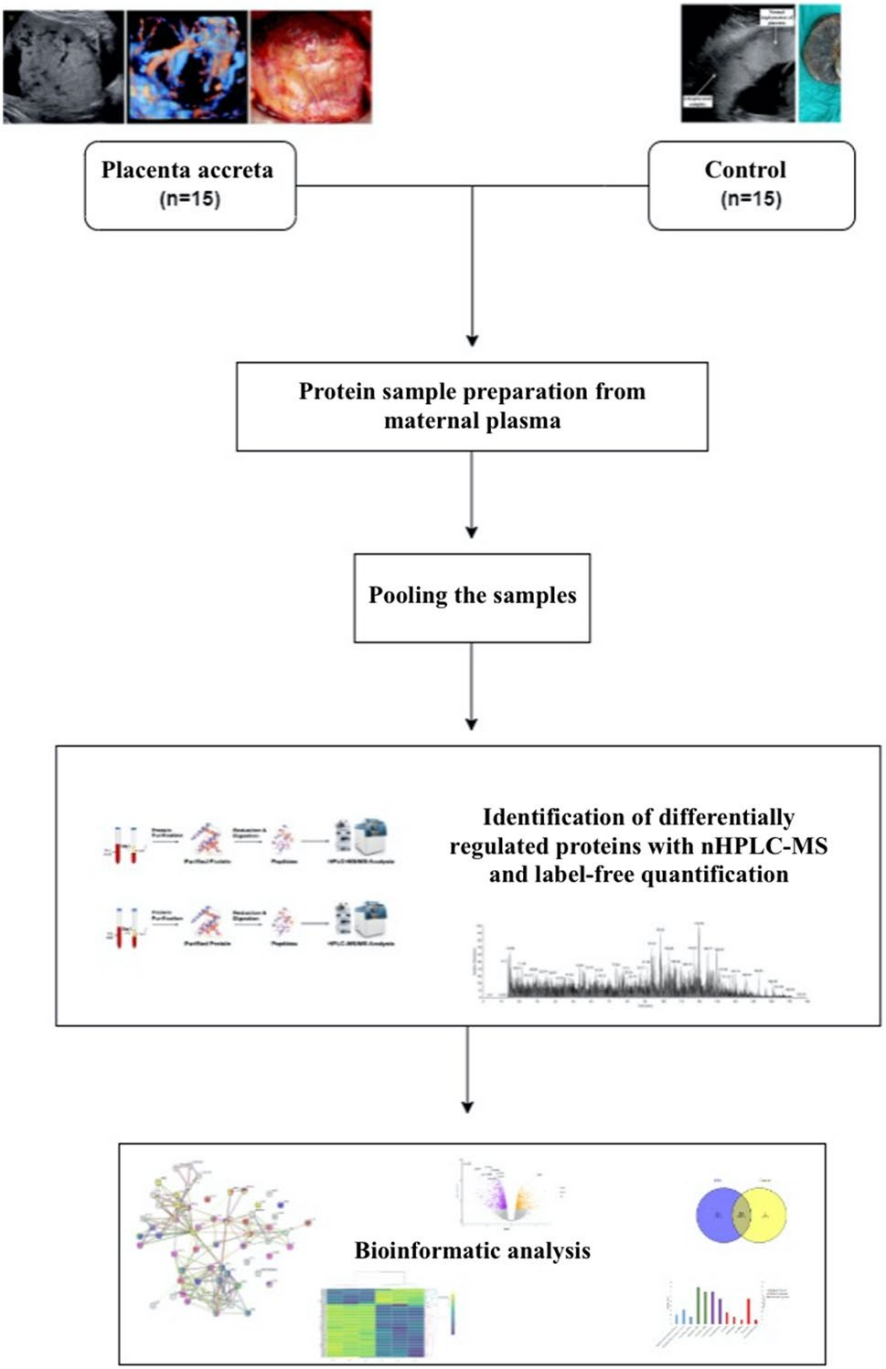
Schematic experimental workflow for steps from preparation of working samples to LC–MS/MS analysis.

During the period of the study, fifteen women were included in the PAS group and 15 women as the control group. Group characteristics are summarized in Table 1. Demographically, mean age (35 ± 4.16 vs. 29.27 ± 5.08, *p* 0.002), number of previous cesarean deliveries (2 [1–3] vs. 1 [0–2], *p* 0.001), parity (2 [1–4] vs. 1 [0–4], *p* 0.002), and cesarean hysteretomy rate (10 [66.7%] vs. 0 [0.0%], *p* 0.001) were significantly higher among the PAS cases compared to control group. Although three cases of urinary complications, all identified as bladder injuries, occurred in the PAS group, the observed urinary complication rate did not reach statistical significance (3 [20.0%] vs. 0 [0.0%], *p* 0.224). Clinically, PAS group was associated with longer postpartum hospitalization compared to control group (5 [3–12] vs. 2 [2–3], *p* < 0.001) (Fig. 1).

**Table 1:** Descriptive statistics on demographic and clinical data of pregnant women included in the study.

	Group		*P* value
	Control group (n = 15)	PAS group (n = 15)	
	Median (range)	Median (range)	
Gestational age at sampling (week)	36 (34–37)	34 (34–36)	0.116*
Gravidity	2 (1–6)	3 (2–7)	0.004*
Parity	1 (0–4)	2 (1–4)	0.002*
Number of previous cesarean deliveries	1 (0–2)	2 (1–3)	0.001*
Gestational age at delivery (week)	36 (35–37)	36 (34–36)	0.133*
Birthweight (g)	2750 (2580–3550)	2760 (2270–3365)	0.436*
Birth length (cm)	47.53 ± 1.36	47.47 ± 1.19	0.887**
1st minute APGAR < 7	7 (7–9)	8 (7–9)	0.148*
5th minute APGAR < 7	9 (8–10)	9 (8–10)	0.806*
Cord blood pH value	7.34 (7.15–7.47)	7.35 (7.26–7.46)	0.683*
Cord blood base excess (mmol/L)	− 3.7 (− 11.1 to 0.3)	− 2.4 (− 13.9 to 0.3)	0.217*
Preoperative hemoglobin value (g/dL)	12.6 (9.1–13.7)	11.3 (10.2–13.5)	0.137*
Preoperative INR value	0.98 (0.8–1.04)	0.99 (0.9–1.05)	0.25*
Duration of operation (min)	35 (30–50)	120 (90–180)	< 0.001*
Estimated blood loss (ml)	200 (120–350)	700 (150–1000)	0.137*
Postpartum hospitalization period (day)	2 (2–3)	5 (3–12)	< 0.001*

	Mean ± SD	Mean ± SD	
Age (year)	29.27 ± 5.08	35 ± 4.16	0.002**
Weight (kg)	77 ± 9.85	79.69 ± 12.48	0.517**
Height (cm)	162.33 ± 4.86	161.07 ± 6.89	0.566**
Body mass index (kg/m^2^)	29.19 ± 3.86	30.64 ± 5.2	0.391**
Preoperative aPTT (s)	26.53 ± 4.68	24.59 ± 3.15	0.195**
Preoperative platelet (× 10^9^/L)	218.87 ± 66.37	211.47 ± 62.49	0.756**
Postoperative 6th hour hemoglobin value (g/dl)	10.91 ± 1.57	9.81 ± 1.47	0.056**

	Number (%)	Number (%)	
Gender			
Female	10 (66.7)	11 (73.3)	1.000***
Male	5 (33.3)	4 (26.7)	
Cesarean hysterectomy	0 (0.0)	10 (66.7)	< 0.001***
Urinary complication	0 (0.0)	3 (20.0)	0.224***
Maternal admission to ICU	0 (0.0)	4 (26.7)	0.100***

Bold values denote statistical significance at the *p* < 0.05 level.

*PAS* placenta accreta spectrum, *SD* standard deviation, *ICU* intensive care unit, *INR* international normalised ratio, *aPTT* activated partial thromboplastin time.

*P value calculated using Mann-Whitney U test; **P value calculated using Independent sample *t* test; ***P value calculated using Fisher-Exact Chi-Square.

**Figure 2:**
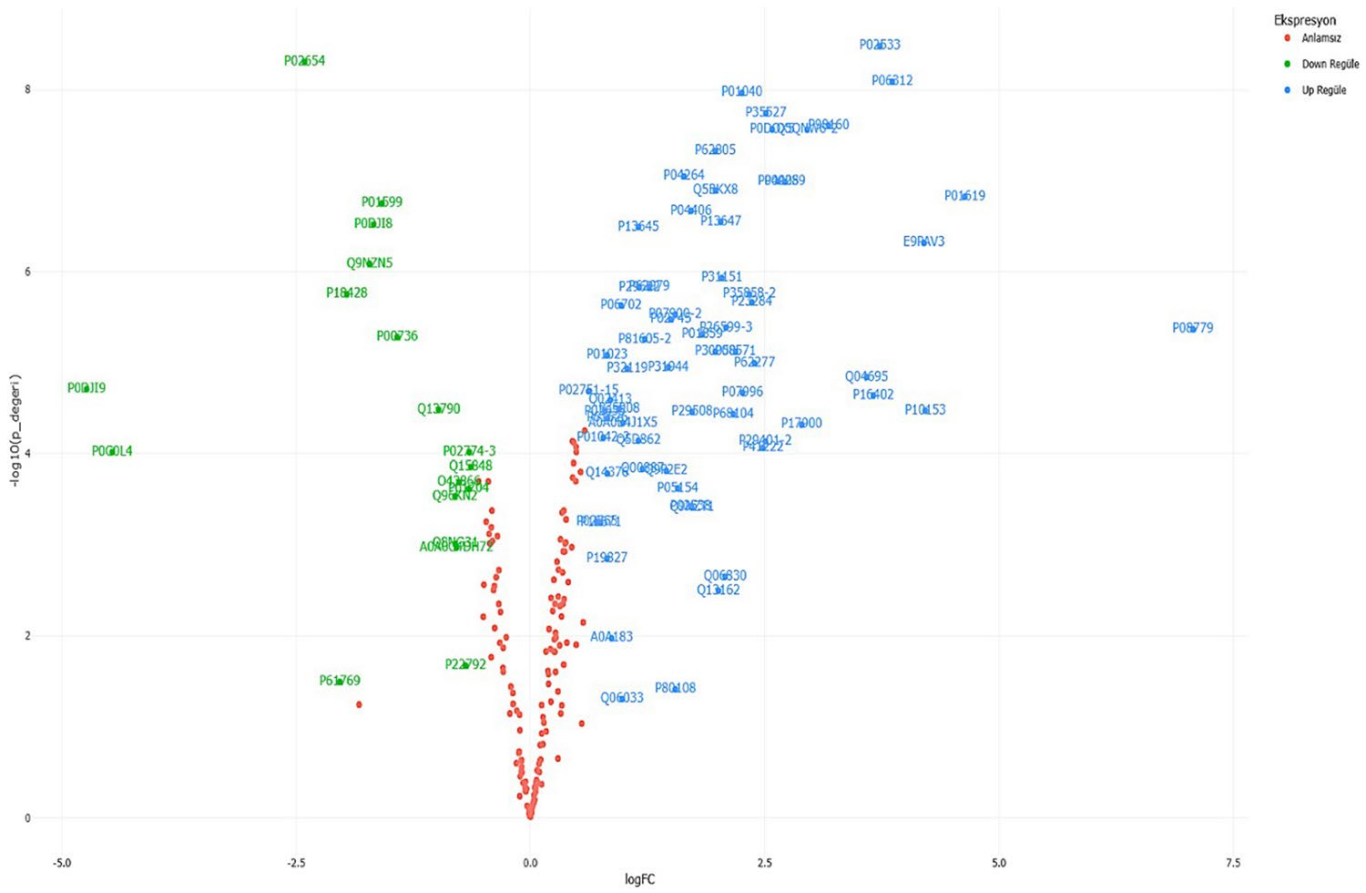
The Volcano plot graph that illustrates the differential regulation of proteins. Red dots represent proteins with no statistically significant difference in expression between the PAS and control groups. Blue dots signify proteins with statistically significant differences between the two groups and higher expression values in the PAS group (up-regulated), while green dots denote proteins with statistically significant differences between the two groups and higher expression values in the control group (down-regulated).

**Figure 3:**
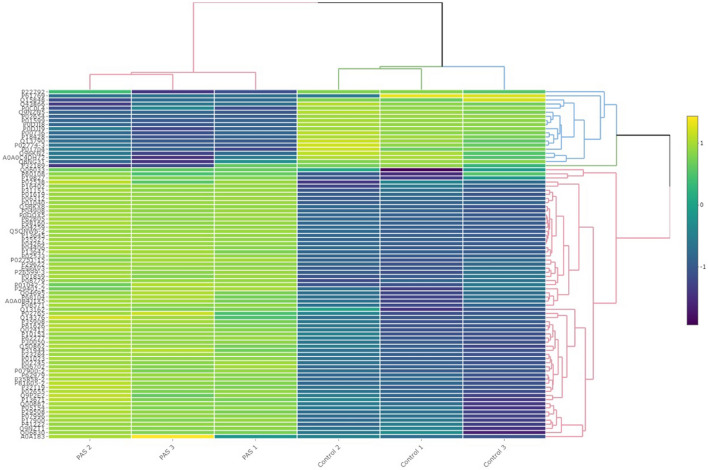
A hierarchical clustered heat map of the relative abundances of proteins in the study and control groups. Darker shades represent lower scaled data values, while lighter shades represent higher scaled data values.

Comparison of proteome differences between PAS and control groups revealed significant alterations in protein expression between the 2 groups. Proteomic analysis identified a total of 228 plasma proteins with detectable expression levels, out of which 85 proteins exhibited statistically significant differences (*P* < 0.001) between PAS cases and control cases (Fig. 2). Among these proteins, 66 were upregulated and 19 were downregulated in PAS cases compared to the control group. A subset of 85 proteins with the most significant differences in expression between cases and controls is presented in Tables 2 and 3. The differential regulation of these proteins is depicted in the Volcano plot, highlighting statistically significant differences across the entire set of regulated proteins (Fig. 2). Hierarchical cluster analysis was performed to classify the groups based on protein levels, and a heat map was generated to visualize the expression patterns (Fig. 3). The analysis clearly distinguished the PAS and control groups, demonstrating distinct fold changes in differentially regulated proteins. Notably, despite some variations among the biological samples, the overall clustering pattern consistently revealed two main sample groups.

**Table 2:** Up-regulated proteins between placenta accreta spectrum cases and control cases.

Protein ID	Protein name	Gene name	PAS mean	PAS SD	Control mean	Control SD	LogFC	FDR
P01859	Immunoglobulin heavy constant gamma 2	IGHG2	20.78	0.09	18.95	0.23	1.83449	< 0.001
P23284	Peptidyl-prolyl cis–trans isomerase B	PPIB	20.9	0.07	18.54	0.28	2.36253	< 0.001
P29622	Kallistatin	SERPINA4	20.46	0.03	19.3	0.09	1.16218	< 0.001
P06312	Immunoglobulin kappa variable 4–1	IGKV4-1	22.73	0.05	18.87	0.16	3.86195	< 0.001
P02538	Keratin type II cytoskeletal 6A	KRT6A	20.89	0.27	19.18	0.44	1.70913	< 0.001
P01619	Immunoglobulin kappa variable 3–20	IGKV3-20	23.33	0.04	18.69	0.37	4.63617	< 0.001
P10153	Non-secretory ribonuclease	RNASE2	22.76	0.07	18.55	0.84	4.21399	< 0.001
P26599	Isoform 3 of Polypyrimidine tract-binding protein 1	PTBP1	20.97	0.07	18.89	0.27	2.08704	< 0.001
P17900	Ganglioside GM2 activator	GM2A	22.11	0.15	19.22	0.6	2.89388	< 0.001
Q9NZT1	Calmodulin-like protein 5	CALML5	20.95	0.05	19.22	0.52	1.72479	< 0.001
P08779	Keratin. type I cytoskeletal 16	KRT16	25.1	0.02	18.03	1.01	7.07055	< 0.001
E9PAV3	Nascent polypeptide-associated complex subunit alpha. muscle-specific form	NACA	21.18	0.08	16.98	0.4	4.19932	< 0.001
Q04695	Keratin. type I cytoskeletal 17	KRT17	21.36	0.27	17.78	0.56	3.58797	< 0.001
P98160	Basement membrane-specific heparan sulfate proteoglycan core protein	HSPG2	22.89	0.11	19.71	0.13	3.18294	< 0.001
P02533	Keratin. type I cytoskeletal 14	KRT14	26.96	0.1	23.23	0.09	3.72985	< 0.001
P41222	Prostaglandin-H2 D-isomerase	PTGDS	20.97	0.06	18.48	0.58	2.48096	< 0.001
P04908	Histone H2A type 1-B/E	HIST1H2AB	24.26	0.07	21.61	0.17	2.64485	< 0.001
P35527	Keratin. type I cytoskeletal 9	KRT9	29.9	0.05	27.39	0.1	2.51254	< 0.001
Q06830	Peroxiredoxin-1	PRDX1	20.15	0.12	18.08	0.88	2.0771	< 0.001
P29401	Isoform 2 of Transketolase	TKT	20.3	0.22	17.8	0.53	2.50552	< 0.001
P04259	Keratin. type II cytoskeletal 6B	KRT6B	26.23	0.06	23.52	0.18	2.71622	< 0.001
P13647	Keratin. type II cytoskeletal 5	KRT5	24.76	0.14	22.73	0.08	2.03099	< 0.001
P62805	Histone H4	HIST1H4A	26.07	0.05	24.1	0.07	1.9727	< 0.001
P16402	Histone H1.3	HIST1H1D	23.7	0.02	20.04	0.69	3.6596	< 0.001
P35858	Isoform 2 of Insulin-like growth factor-binding protein complex acid labile subunit	IGFALS	21.02	0.11	18.69	0.25	2.3364	< 0.001
Q5QNW6	Isoform 2 of Histone H2B type 2-F	HIST2H2BF	24.9	0.02	21.95	0.15	2.95295	< 0.001
P80108	Phosphatidylinositol-glycan-specific phospholipase D	GPLD1	19.37	0.22	17.82	1.25	1.54757	< 0.001
P62277	40S ribosomal protein S13	RPS13	21.52	0.02	19.13	0.38	2.39112	< 0.001
P01040	Cystatin-A	CSTA	23.41	0	21.16	0.07	2.25607	< 0.001
P08571	Monocyte differentiation antigen CD14	CD14	20.65	0.08	18.46	0.32	2.1884	< 0.001
P68104	Elongation factor 1-alpha 1	EEF1A1	21.44	0.18	19.28	0.39	2.1654	< 0.001
P30050	60S ribosomal protein L12	RPL12	22.73	0.06	20.75	0.29	1.97376	< 0.001
Q5BKX8	Caveolae-associated protein 4	CAVIN4	25.04	0.04	23.06	0.12	1.97532	< 0.001
P07996	Thrombospondin-1	THBS1	21.16	0.08	18.89	0.41	2.26357	< 0.001
P04264	Keratin. type II cytoskeletal 1	KRT1	30.83	0.03	29.19	0.07	1.63986	< 0.001
P02745	Complement C1q subcomponent subunit A	C1QA	24.05	0.05	22.56	0.17	1.49755	< 0.001
P81605	Isoform 2 of Dermcidin	DCD	24.15	0.1	22.93	0.11	1.22032	< 0.001
P0DOX5	Immunoglobulin gamma-1 heavy chain		24.21	0.06	21.63	0.11	2.57977	< 0.001
P31151	Protein S100-A7	S100A7	23.46	0.07	21.42	0.2	2.04286	< 0.001
P07900	Isoform 2 of Heat shock protein HSP 90-alpha	HSP90AA1	22.01	0.11	20.46	0.14	1.54478	< 0.001
P04406	Glyceraldehyde-3-phosphate dehydrogenase	GAPDH	24.62	0.07	22.9	0.09	1.71432	< 0.001
P29508	Serpin B3	SERPINB3	21.24	0.13	19.51	0.31	1.72562	< 0.001
P13671	Complement component C6	C6	24.16	0.08	23.41	0.21	0.751649	< 0.001
P31944	Caspase-14	CASP14	21.67	0.15	20.2	0.17	1.47087	< 0.001
Q9P2E2	Kinesin-like protein KIF17	KIF17	21.31	0.2	19.87	0.31	1.4488	< 0.001
P62979	Ubiquitin-40S ribosomal protein S27a	RPS27A	24	0.07	22.73	0.09	1.26772	< 0.001
Q5D862	Filaggrin-2	FLG2	20.6	0.09	19.45	0.23	1.15151	< 0.001
P13645	Keratin. type I cytoskeletal 10	KRT10	30.56	0.01	29.4	0.04	1.15254	< 0.001
Q00887	Pregnancy-specific beta-1-glycoprotein 9	PSG9	20.41	0.07	19.22	0.29	1.19618	< 0.001
Q13162	Peroxiredoxin-4	PRDX4	21.7	0.07	19.69	0.92	2.00611	< 0.001
P06702	Protein S100-A9	S100A9	25.83	0.04	24.86	0.06	0.96675	< 0.001
A0A0B4J1X5	Immunoglobulin heavy variable 3–74	IGHV3-74	22.81	0.07	21.82	0.17	0.986614	< 0.001
A0A183	Late cornified envelope protein 6A	LCE6A	17.5	0.48	16.63	0.17	0.86783	< 0.001
P35908	Keratin. type II cytoskeletal 2 epidermal	KRT2	28.83	0.07	27.88	0.15	0.947344	< 0.001
P01023	Alpha-2-macroglobulin	A2M	30.97	0.04	30.16	0.08	0.817748	< 0.001
Q02413	Desmoglein-1	DSG1	21.85	0.05	20.99	0.12	0.852002	< 0.001
Q14376	UDP-glucose 4-epimerase	GALE	24.45	0.17	23.64	0.09	0.819099	< 0.001
P32119	Peroxiredoxin-2	PRDX2	23.23	0.09	22.19	0.11	1.03265	< 0.001
P01042	Isoform LMW of Kininogen-1	KNG1	27.18	0.08	26.4	0.12	0.778082	< 0.001
P61626	Lysozyme C	LYZ	22.83	0.07	22.01	0.12	0.81999	< 0.001
P02655	Apolipoprotein C-II	APOC2	29.03	0.06	28.24	0.11	0.794366	< 0.001
P05154	Plasma serine protease inhibitor	SERPINA5	21.77	0.08	20.2	0.43	1.57285	< 0.001
P02765	Alpha-2-HS-glycoprotein	AHSG	27.24	0.14	26.53	0.16	0.709687	< 0.001
P02751	Isoform 15 of Fibronectin	FN1	25.24	0.03	24.62	0.05	0.621365	< 0.001
Q06033	Inter-alpha-trypsin inhibitor heavy chain H3	ITIH3	22.25	0.03	21.27	0.86	0.978899	< 0.001
P19827	Inter-alpha-trypsin inhibitor heavy chain H1	ITIH1	26.38	0.05	25.56	0.3	0.81678	< 0.001

*PAS* placenta accreta spectrum, *SD* standard deviation, *FC* fold change, *FDR* false discovery rate.

**Table 3:** Down-regulated proteins between placenta accreta spectrum cases and control cases.

Protein ID	Protein name	Gene name	PAS mean	PAS SD	Control mean	Control SD	LogFC	FDR
P32189	Glycerol kinase	GK	19.7	1.63	21.53	0.44	− 1.8282	< 0.001
P18428	Lipopolysaccharide-binding protein	LBP	20.42	0.16	22.37	0.15	− 1.9578	< 0.001
P02774	Isoform 3 of Vitamin D-binding protein	GC	29.34	0.07	30	0.1	− 0.65129	< 0.001
Q15848	Adiponectin	ADIPOQ	23.52	0.08	24.15	0.1	− 0.63696	< 0.001
P01704	Immunoglobulin lambda variable 2–14	IGLV2-14	21.6	0.13	22.26	0.1	− 0.6587	< 0.001
O43866	CD5 antigen-like	CD5L	24.29	0.17	25.05	0.07	− 0.76158	< 0.001
A0A0C4DH72	Immunoglobulin kappa variable 1–6	IGKV1-6	21.09	0.24	21.88	0.14	− 0.79015	< 0.001
P00736	Complement C1r subcomponent	C1R	20.75	0.14	22.17	0.13	− 1.42028	< 0.001
P22792	Carboxypeptidase N subunit 2	CPN2	22.82	0.47	23.51	0.07	− 0.69241	< 0.001
Q96KN2	Beta-Ala-His dipeptidase	CNDP1	18.67	0.16	19.48	0.14	− 0.80517	< 0.001
Q8NG31	Kinetochore scaffold 1	KNL1	19.13	0.27	19.93	0.03	− 0.80089	< 0.001
Q13790	Apolipoprotein F	APOF	23.26	0.08	24.24	0.15	− 0.97758	< 0.001
P01599	Immunoglobulin kappa variable 1–17	IGKV1-17	20.53	0.06	22.12	0.06	− 1.58419	< 0.001
P0DJI8	Serum amyloid A-1 protein	SAA1	24.73	0.1	26.4	0.07	− 1.67552	< 0.001
P02654	Apolipoprotein C-I	APOC1	24.03	0.04	26.44	0.04	− 2.4085	< 0.001
P0DJI9	Serum amyloid A-2 protein	SAA2	18.23	0.86	22.97	0.14	− 4.73552	< 0.001
P0C0L4	Complement C4-A	C4A	18.5	1.07	22.96	0.11	− 4.46277	< 0.001
P61769	Beta-2-microglobulin	B2M	18.4	0.06	20.44	1.58	− 2.03388	< 0.001
Q9NZN5	Rho guanine nucleotide exchange factor 12	ARHGEF12	18.41	0.08	20.13	0.14	− 1.71546	< 0.001

*PAS* placenta accreta spectrum, *SD* standard deviation, *FC* Fold change, *FDR* false discovery rate.

To construct a robust classification model, a variable selection technique called Random Forest Recursive Feature Elimination (RF-RFE) was employed. This method aimed to optimize model performance by identifying a subset of informative proteins from the initial set of 228 proteins. Through comprehensive bioinformatic analysis, a refined set of 20 proteins was selected for subsequent model construction (Table 4). Evaluation of performance metrics, including accuracy, for various models (random forest, decision trees, and logistic regression) using the selected proteins revealed that a specific model exhibited the highest classification performance. This model demonstrated an impressive accuracy of 96.9% (range: 96.0–97.7%), outperforming the other models in accurately classifying the target groups (Table 5).

**Table 4:** Comparison of 20 proteins selected by Random Forest Recursive Feature Elimination (RF-RFE) method.

Protein ID	Control group	PAS group	*P* value
P01859	18.9453 ± 0.2311	20.7798 ± 0.0941	< 0.001
P23284	18.535 ± 0.278	20.8975 ± 0.0668	< 0.001
P29622	19.301 ± 0.092	20.4632 ± 0.033	< 0.001
P06312	18.8697 ± 0.1638	22.7316 ± 0.0522	< 0.001
P02538	19.1833 ± 0.4417	20.8925 ± 0.2682	0.0046
P01619	18.6937 ± 0.3677	23.3298 ± 0.0419	< 0.001
P10153	18.548 ± 0.845	22.762 ± 0.0701	0.001
P26599-3	18.887 ± 0.2724	20.974 ± 0.0657	< 0.001
P17900	19.2187 ± 0.5974	22.1125 ± 0.1456	0.0012
Q9NZT1	19.2247 ± 0.5225	20.9495 ± 0.048	0.0047
P08779	18.0293 ± 1.0098	25.0999 ± 0.0228	< 0.001
E9PAV3	16.9785 ± 0.4009	21.1778 ± 0.0791	< 0.001
P98160	19.7079 ± 0.1267	22.8908 ± 0.1115	< 0.001
P02533	23.2271 ± 0.0898	26.9569 ± 0.0973	< 0.001
P41222	18.4843 ± 0.5792	20.9652 ± 0.0572	0.0018
P04908	21.6134 ± 0.1668	24.2582 ± 0.0654	< 0.001
Q06830	18.0777 ± 0.8791	20.1548 ± 0.1192	0.0154
P29401-2	17.7958 ± 0.526	20.3013 ± 0.2233	0.0016
P04259	23.5174 ± 0.1753	26.2336 ± 0.062	< 0.001
P13647	22.727 ± 0.08	24.758 ± 0.1353	< 0.001
P62805	24.1007 ± 0.0732	26.0734 ± 0.0537	< 0.001
P16402	20.0361 ± 0.6876	23.6957 ± 0.0183	< 0.001
P62277	19.1261 ± 0.3828	21.5172 ± 0.0151	< 0.001
P30050	20.7528 ± 0.2874	22.7266 ± 0.0647	< 0.001
Q9P2E2	19.8654 ± 0.3064	21.3142 ± 0.2026	0.0024

*PAS* placenta accreta spectrum.

**Table 5:** Values for metrics of classification performance of random forest, decision trees, and logistic regression models.

Models	Metric	Value (%) (95% confidence interval)
Random forest	Accuracy	96.9 (96.0–97.7)
	Specificity	92.4 (90.1–94.4)
	Sensitivity	100 (99.6–100)
	G-mean	97.4 (96.6–98.2)
	MCC	93.7 (92.5–94.9)
	F1 score	97.4 (96.6–98.2)
Decision tree	Accuracy	79.1 (77.1–81.1)
	Specificity	65.7 (62.1–69.2)
	Sensitivity	89.6 (87.4–91.5)
	G-mean	80.0 (78.0–82.0)
	MCC	57.7 (55.2–60.1)
	F1 score	82.8 (80.9–84.6)
Logistic regression	Accuracy	87.0 (85.3–88.7)
	Specificity	74.6 (71.5–77.7)
	Sensitivity	100 (99.5–100)
	G-mean	88.9 (87.3–90.5)
	MCC	76.8(74.7–78.9)
	F1 score	88.3 (86.7–89.9)

*MCC* Matthew’s correlation coefficient.

**Figure 4:**
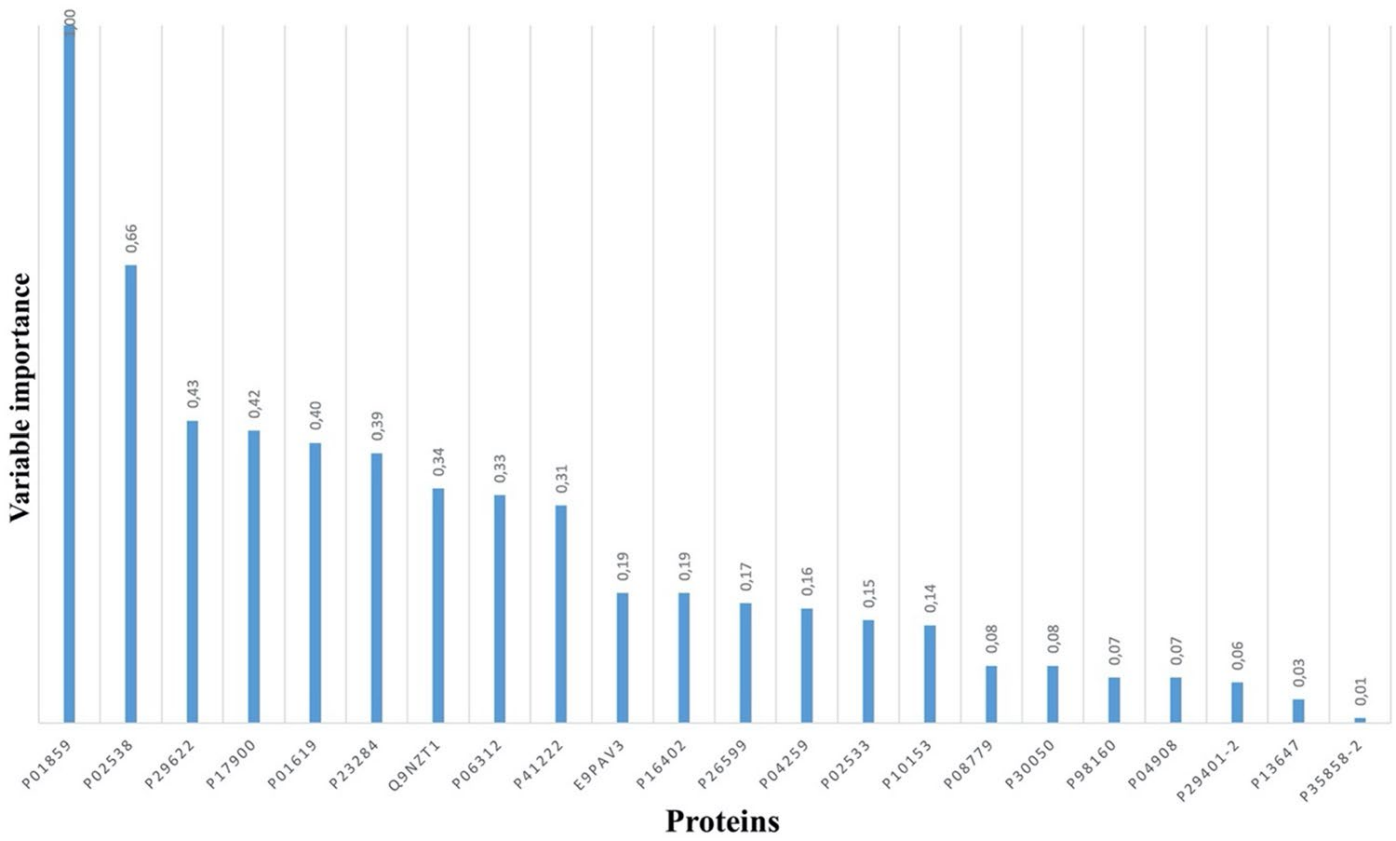
The importance values for potential biomarkers.

Based on the variable significance values obtained from the model, certain proteins displayed notable associations. Specifically, the proteins encoded by P01859 [Immunoglobulin heavy constant gamma 2 (IGHG2)], P02538 [Keratin type II cytoskeletal 6A (K6A)], P29622 [Kallistatin (also known as Serpin A4)], P17900 [Ganglioside GM2 activator Calmodulin-like protein 5 (GM2AP) (also known as cerebroside sulfate activator protein, shingolipid activator protein 3)], and P01619 [Immunoglobulin kappa variable 3–20 (IGKV3-20)] exhibited high significance values (> 0.4), indicating their relevance in discriminating between PAS and control groups (Fig. 4). Further bioinformatic analyses unveiled distinct expression patterns of these five proteins between the PAS and control groups. Notably, they exhibited fold changes approximately 1.83, 1.70, 1.16, 2.89, and 4.63 times higher, respectively, in the PAS group compared to the control group. These findings provide insights into the potential functional implications and differential expression profiles of these proteins in the context of the investigated condition.

**Figure 5:**
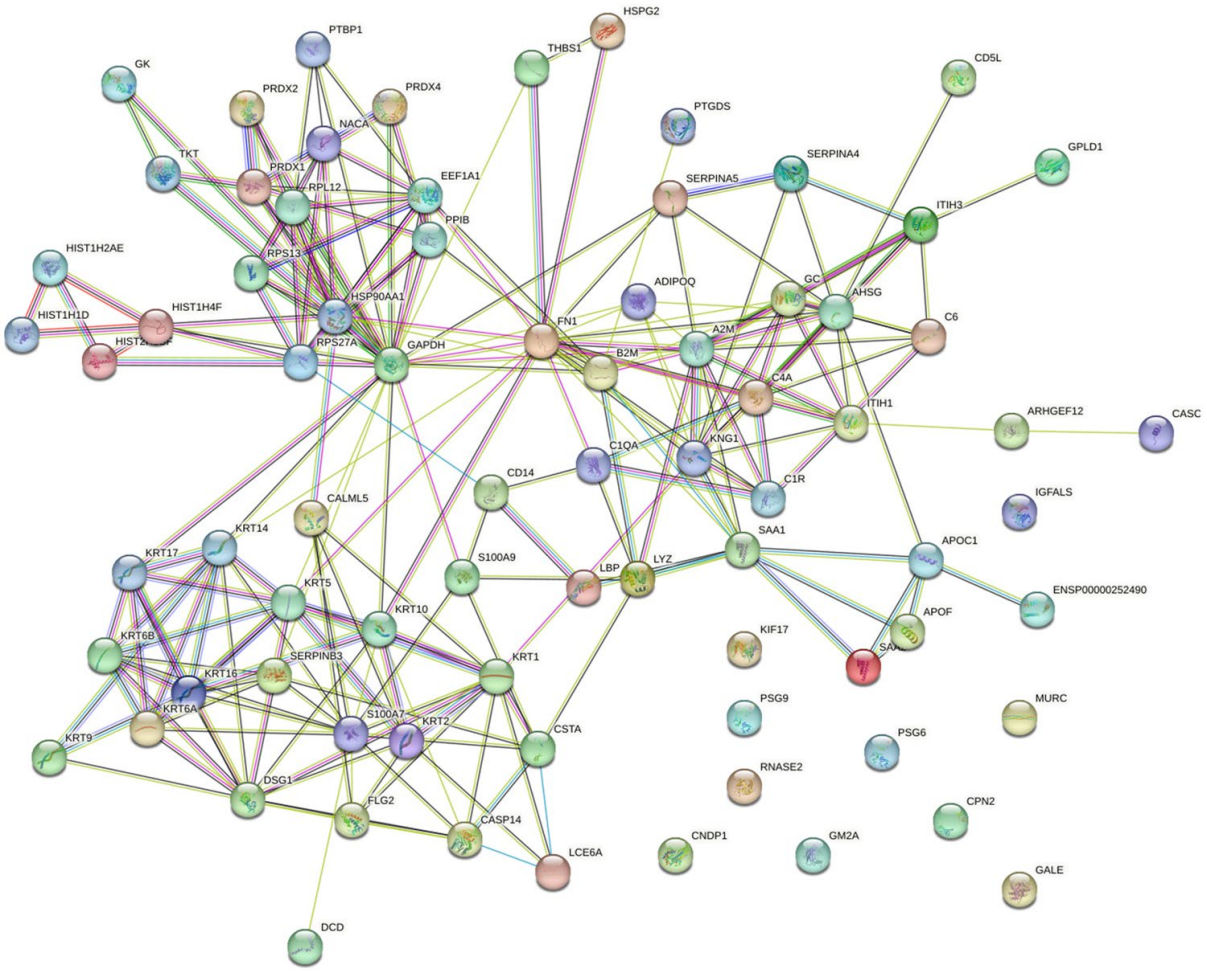
Protein interaction network created by STRING using the upregulated and downregulated proteins in the PAS group. Color code for edge interpretation: neighborhood (green), gene fusion (red), cooccurrence (blue), coexpression (dark), experiments (pink), databases (light blue), textmining (yellow), and homology (purple).

To gain a comprehensive understanding of the biological functions of the upregulated and downregulated proteins identified through LC–MS/MS analysis, we conducted a systematic investigation into their roles in the context of PAS. Employing Panther classification analysis, we analyzed the 85 proteins discovered in our study to identify the enriched signaling pathways and biological processes influenced by these PAS-associated proteins. To explore the interconnections and interactions among these proteins, we utilized the STRING version online analysis tool, which provided valuable information regarding protein associations, upstream regulators, and downstream biological effects (Fig. 5).

## Discussion

PAS, also known as abnormally invasive placenta, is a significant and increasingly prevalent complication in the field of obstetrics. This condition poses substantial risks to both maternal and fetal/neonatal outcomes, including risks of maternal morbidity and mortality, increased likelihood of preterm birth, low birth weight infants, and perinatal mortality^7^. Standard antenatal or preoperative preparations, including multidisciplinary management within PAS-specialized centers, have been adopted to improve maternal and neonatal outcomes, for suspected cases prior to delivery^8^. Such care should be supported by efficient approaches that rely on understanding disease anatomy and pathogenesis to achieve early and accurate antenatal suspicion of women with PAS.

The exact pathogenesis of PAS remains unclear, although the prevailing theory suggests that prior uterine surgeries involving the endometrial-myometrial interface may lead to decidualization defect in areas of uterine scarring. This decidualization process, in turn, facilitates abnormal attachment of placental villi to the myometrium, along with increased trophoblast invasion^9^. Obstetric ultrasonography currently serves as the primary diagnostic method for prenatal identification of PAS. However, definitive prenatal diagnosis of PAS cannot be established solely through imaging techniques. Consequently, the development of an improved diagnostic model for early and accurate detection of PAS is imperative.

Several maternal biomarkers have been associated with PAS. Studies have reported elevated levels of maternal serum alpha-fetoprotein (AFP) in PAS cases compared to normal pregnancies, often exhibiting a change of 2–2.5 times the median value in the second trimester^10^. Secretory placental hormones, such as human chorionic gonadotropin (hCG) and its free beta-subunit (β-hCG), as well as pregnancy-associated plasma protein A (PAPP-A), have also been linked to the development of PAS around the 12th week of pregnancy. Notably, hCG levels tend to be lower in the maternal serum of PAS cases, while PAPP-A concentrations are elevated^11^. However, the clinical utility of these hormones in diagnosis of PAS is limited because of their poor sensitivity and specificity. These hormones are generally associated with various genetic disorders, such as trisomy 21.

In the current study, utilizing bioinformatics analysis on experimental data obtained from proteomic analyses of maternal serum samples, we identified significant upregulation of specific proteins in the PAS group compared to the control group. Specifically, the protein encoded by P08779, known as "Keratin type I cytoskeletal 16," exhibited 7.07 times higher expression in the PAS group. Similarly, the protein encoded by E9PAV3, designated as "Nascent polypeptide-associated complex subunit alpha muscle-specific form," demonstrated 4.19 times higher expression. Additionally, the proteins coded by P10153 ("Non-secretory ribonuclease") and P01619 ("Immunoglobulin kappa variable 3–20") displayed 4.21- and 4.63-times higher expression, respectively, in the PAS group. Similarly, Shainker et al. employed an aptamer-based proteomics platform to analyze plasma samples, focusing on alterations in 1305 unique proteins. Among the top 50 dysregulated proteins identified in participants with PAS, a notable proportion comprised inflammatory cytokines, factors involved in vascular remodeling regulation, and extracellular matrix proteins associated with invasion regulation. Notably, the authors found that the use of the top 21 proteins distinctly differentiated PAS cases from control cases. To further validate their findings, the researchers utilized enzyme-linked immunosorbent assay and confirmed dysregulation of four proteins in PAS compared to control cases: antithrombin III, plasminogen activator inhibitor 1 concentrations, soluble Tie2, and soluble vascular endothelial growth factor receptor 2. The differences between the results of Shainker et al.’s study and our own findings may be attributed to several factors. Firstly, the disparity in sample size and heterogeneity of the study population may have contributed to variations in the observed protein dysregulation patterns. Additionally, Shainker et al.^12^ were unable to access ultrasound data from the participants, which may have affected the accuracy of their assessments. Furthermore, the researchers were unable to histopathologically confirm all cases of PAS, potentially leading to misdiagnosis or incomplete characterization of the condition. These discrepancies highlight the importance of conducting larger-scale studies with well-defined and homogeneous populations, incorporating comprehensive clinical data and histopathological confirmation to enhance the accuracy and reliability of findings. By addressing these limitations, future investigations can further elucidate the protein dysregulation profiles associated with PAS, ultimately advancing our understanding of the condition and its diagnostic potential.

Our comprehensive analysis revealed that several proteins identified in our study are intricately involved in critical processes such as invasion, angiogenesis, vascularization, and epithelial-mesenchymal transition, all of which have established roles in the pathogenesis of PAS. These findings provide valuable insights into the specific molecular components and signaling pathways underlying abnormal placentation. Furthermore, our results suggest that a panel of plasma proteins holds promise as potential biomarkers for the identification and monitoring of individuals with PAS, facilitating early detection and improved management of this complex condition. In parallel to our study, Chen et al. conducted an integrated analysis of transcriptomic and proteomic sequencing data from placenta tissues obtained from five patients with PAS and five healthy pregnant women as controls. Their analysis identified a total of 728 differentially expressed messenger RNAs and 439 differentially expressed proteins between the PAS group and the non-PAS group. Among these, 23 hub genes were found to be differentially expressed at both the transcriptome and proteome levels. Functional enrichment analysis of these differentially expressed genes revealed their involvement in crucial biological processes such as cell proliferation, migration, and vascular development^13^. Taken together, our study and the findings of Chen et al. contribute to the growing body of knowledge regarding the molecular mechanisms underlying PAS. The identification of dysregulated proteins and genes associated with key processes in placental pathology provides a foundation for further investigation and potential therapeutic targets. However, it is important to note that additional studies with larger sample sizes and diverse populations are needed to validate these findings and establish their clinical utility in the diagnosis, prognosis, and management of PAS.

In normal placentation, invasive trophoblast cells precisely regulate their invasion within a specific myometrial region through intricate interactions with maternal blood vessels. Dysregulation of this process can lead to the development of PAS; however, the underlying molecular pathways are not yet well understood. Our study highlighted the upregulation of kallistatin (P29622), an endogenous protein with dual roles in angiogenesis, apoptosis, and oxidative stress. The structural elements of kallistatin, namely the active site and heparin-binding domain, play a critical role in regulating distinct signaling pathways and biological functions^14^. Mechanistically, the active site of kallistatin is key in stimulating the proliferation, migration, adhesion, and tube formation of endothelial progenitor cells by activating Akt-eNOS signaling and increasing vascular endothelial growth factor levels. Endothelial progenitor cells act as a continuous source of replenishment for damaged blood vessels by enhancing neovascularization in response to endothelial injury^15^. Excessive expression of kallistatin may contribute to the development of PAS by disrupting blood vessel formation and regulating the activation of the Akt-eNOS signaling pathway.

Immunoglobulins, also known as antibodies, are glycoproteins produced by B lymphocytes and play critical roles in humoral immunity. During the recognition phase of humoral immunity, membrane-bound immunoglobulins serve as receptors that, upon binding to a specific antigen, trigger the clonal expansion and differentiation of B lymphocytes into immunoglobulin-secreting plasma cells. Secreted immunoglobulins then mediate the effector phase of humoral immunity, resulting in the elimination of bound antigens ^16^. Upregulated levels of immunoglobulin heavy constant gamma 2 (IGHG2) and immunoglobulin kappa variable 3–20 (IGKV3-20), functional isoforms of IgG, can alter the cytolytic activity of immune effector cells. Although no study has specifically investigated the role of these proteins as diagnostic or prognostic biomarkers in any disease, it has been observed that IGHG1 overexpression accelerates malignant cell migration and invasion in vitro and is associated with lymph node metastasis in ovarian cancer^17^. These findings suggest that vascular inflammation mediated by these proteins could contribute to the development of abnormally invasive placenta.

Keratin, a prominent constituent of intermediate filament proteins, is primarily expressed in epithelial tissues. Previous research has indicated that keratin not only plays a role in cellular protection against non-mechanical stress and impairment but also contributes to the regulation of cell growth and apoptosis. Within the family of keratin proteins, Keratin 6A (KRT6A) holds particular significance in the regulation of epithelial migration and maintaining tissue integrity^18^. A study conducted by Chen et al. demonstrated that silencing KRT6A led to a decrease in the expression of matrix metalloproteinase (MMP)-2 and MMP-9, while simultaneously promoting the expression of tissue inhibitor of metalloproteinases 2 in nasopharyngeal carcinoma cells^19^. These findings suggest a potential modulatory role of KRT6A in the invasion and metastasis processes associated with malignant diseases. Additionally, KRT6A may contribute to invasion and epithelial-mesenchymal transition, both of which are known to be involved in the pathogenesis of PAS.

GM2AP is an essential cofactor for the degradation of GM2 ganglioside to GM3 by lysosomal hexaminidase A. Aberrant expression of GM2AP is related to tumor-associated gangliosides involved in cancer progression and plays a role in the induction of invasion and metastasis^20^. Gangliosides synthesized by tumor cells and shed into the microenvironment have been shown to suppress the antitumor immune response, including natural killer cell cytotoxicity, as demonstrated in numerous studies^21^. These findings suggest that vascular inflammation mediated by this protein could contribute to the development of abnormally invasive placenta.

In summary, our proteomic analysis of PAS cases identified dysregulated proteins associated with key biological functions such as invasion, angiogenesis, inflammation, and coagulation. These findings provide valuable insights into the molecular mechanisms underlying abnormal placentation and suggest potential targets for further research and the development of diagnostic or therapeutic strategies for PAS.

This study possesses several notable strengths that enhance its scientific rigor and validity. Firstly, the utilization of prospectively collected specimens ensured the availability of well-phenotyped samples, enabling robust and reliable data analysis. Moreover, the inclusion of a several proteins in an unbiased manner through comprehensive testing contributes to a comprehensive understanding of the molecular landscape associated with the studied condition. Additionally, the fact that all participants were diagnosed and managed within a single center minimizes the potential confounding effects of intercenter heterogeneity, particularly in terms of preoperative and peroperative diagnosis, thus enhancing the internal validity of the findings.

However, certain limitations should be considered when interpreting the results of this study. First and foremost, the small sample size might restrict the generalizability of the findings, warranting caution in extrapolating the results to larger populations. Additionally, the absence of an independent cohort to validate the identified proteins hinders the external validity and generalizability of the findings. Furthermore, it is important to acknowledge that the results obtained from this study were not validated using an independent and validated enzyme-linked immunosorbent assay method for the five dysregulated proteins. Therefore, future research should aim to validate these proteins using robust validation techniques to ensure the reliability and reproducibility of the findings. Another limitation to consider is that many of the proteins identified in this study are known to change with gestational age during normal pregnancy. While our study design meticulously incorporated a well-matched control group for gestational age, intending to mitigate variations associated with different stages of pregnancy, we also proactively addressed potential confounding factors such as maternal age, comorbidities, and other demographic variables through stringent inclusion and exclusion criteria. It is crucial to acknowledge that, despite our efforts to account for specific confounding factors, the diagnostic potential of the identified proteins may still be influenced by variables not explicitly explored in this investigation. Future research and validation studies in larger cohorts, encompassing diverse patient populations and clinical settings, will be essential for a comprehensive understanding of the diagnostic utility and potential limitations of the identified plasma proteins as biomarkers for PAS.

Following extensive bioinformatic analyses in this study, a variable selection method was employed to identify proteins with differential expression between two distinct groups. Subsequently, machine learning methods were utilized to classify these selected proteins, with the Random Forest algorithm employed specifically to classify PAS. In addition, the variable significance of these proteins was determined. Based on the outputs of these analyses, it is hypothesized that five proteins (P01859, P02538, P29622, P17900, P01619) may serve as potential biomarkers that could aid clinicians in the early diagnosis of PAS. However, further investigations are warranted to validate and evaluate the diagnostic and prognostic value of these candidate proteomic biomarkers in dedicated PAS cohorts. Rigorous testing in appropriate study populations is crucial to establish the clinical utility, sensitivity, specificity, and predictive power of these protein markers in the context of diagnosing PAS and assessing disease prognosis. By conducting follow-up studies that involve larger sample sizes and diverse populations, the validity, and potential applications of these identified protein biomarkers in PAS diagnosis can be robustly established. Such work would contribute to advancing personalized healthcare approaches and improve management in women with PAS.

## Methods

We enrolled all pregnant women diagnosed with PAS who delivered at the Department of Obstetrics and Gynecology, Inonu University Faculty of Medicine, between 01/04/2021 and 01/01/2023, meeting the following inclusion criteria: women aged 18–39 years, with singleton viable pregnancies, diagnosed with PAS, and delivering at our investigating hospital. Exclusion criteria included women with multiple pregnancies, pregestational diabetes mellitus, chronic hypertension, or concomitant systemic maternal diseases (such as dyslipidemia, chronic renal insufficiency, malignancies, pulmonary, or cardiac diseases). Pregnancies with fetuses exhibiting abnormal karyotype or malformations, or complicated by preeclampsia, gestational diabetes, premature rupture of membranes, or cholestasis of pregnancy were also excluded. A control group comprising women receiving care at the same hospital during the study period, without a diagnosis of PAS, was recruited and matched to the PAS group based on gestational age. Gestational age in all participants was confirmed through first-trimester ultrasound measurements. Eligible women were consented prenatally at the time of diagnosis of PAS, and an informed consent was obtained on approval to participate.

The prenatal diagnosis of PAS was based on grayscale, color, and three-dimensional power Doppler ultrasound findings, in conjunction with intraoperative characteristics including failure of the placenta to separate and fragmentation. Histopathological confirmation of the diagnosis was obtained from patients undergoing hysterectomy or local resection. Maternal blood samples were collected within 1 week prior to delivery, using appropriate tubes. After allowing a 30-min clotting period at room temperature, plasma samples were obtained by centrifugation at 1500×*g* for 10 min and subsequently stored at − 80 °C until analysis. Upon reaching the desired sample size, the plasma samples were thawed, and proteomic analyses were conducted.

### Proteomic analysis

Immunoglobulin removal from serum samples was achieved using the Bio-rad NGS chromatography system. Purified serum samples, depleted of immunoglobulins through protein G column purification, were mixed with 6X lamellar loading dye and heated at 60 °C for 15 min, followed by cooling on ice and loading into PrepCell. This step was crucial for protein denaturation. Gel continuity parameters, including gradient, gradual, and constant features, as well as height and concentration, were optimized using the PrepCell system. Enzymatic digestion of protein sections for liquid chromatography-mass spectrometry/mass spectrometry (LC–MS/MS) analysis was performed following the protocol provided by the Thermo Scientific kit (#89895). The obtained data were analyzed using Proteome Discoverer 2.2 software (Thermo Scientific, USA) for protein identification.

### Statistical and bioinformatics analysis

Quantitative data were presented as mean ± standard deviation or median and range, while qualitative data were expressed as numbers (percentage). The normal distribution of data was assessed using the Shapiro–Wilk test. Two-Sample T-test and Mann–Whitney U-test were employed, as appropriate, to evaluate intergroup differences in quantitative variables. The relationship between qualitative variables and group variables was examined using the Chi-square test. A *p* value less than 0.05 was deemed statistically significant. Unsupervised hierarchical clustering was applied to classify groups based on protein levels, and protein expressions were visualized using a heatmap. Random Forest, Logistic Regression, and Decision Trees methods were employed for classification purposes. Bioinformatics analysis, utilizing Panther classification analysis (http://pantherdb.org/), was employed to classify proteins based on biological process, cellular component, and molecular function. Protein–protein interaction networks and potential pathways associated with the identified differentially regulated proteins (DRPs) were obtained using the online analysis tool search tool for the retrieval of interacting genes/proteins (STRING) version 11.5 (string-db.org/), which encompasses known and predicted physical and functional protein–protein interactions. Lower false discovery rate (FDR) values indicate greater importance of the identified processes and pathways. A schematic workflow outlining the steps from sample preparation to LC–MS/MS analysis is presented in Fig. 1.

### Ethics statement

The present study received approval from the Clinical Research Ethics Committee of Inonu University (Approval number: 2021/81), and the investigators strictly adhered to the principles outlined in the World Medical Association's Declaration of Helsinki, incorporating the modifications introduced in 2013. All participating women were provided with both written and verbal information about the study, and informed consent was subsequently obtained.

## Data Availability

The datasets generated during the current study are available from the corresponding author on reasonable request.
